# IoT Intrusion Detection Taxonomy, Reference Architecture, and Analyses

**DOI:** 10.3390/s21196432

**Published:** 2021-09-26

**Authors:** Khalid Albulayhi, Abdallah A. Smadi, Frederick T. Sheldon, Robert K. Abercrombie

**Affiliations:** 1 Department of Computer Science, University of Idaho, Moscow, ID 83844, USA; Sheldon@ieee.org; 2 Department of ECE, Science, University of Idaho, Moscow, ID 83844, USA; smad4875@vandals.uidaho.edu; 3 Oak Ridge National Laboratory, Oak Ridge, TN 37830, USA; abercrombier@ieee.org

**Keywords:** anomaly-based IDS, IoT architecture mapping, deep learning, machine learning (ML), intrusion-detection systems (IDS), IoT security

## Abstract

This paper surveys the deep learning (DL) approaches for intrusion-detection systems (IDSs) in Internet of Things (IoT) and the associated datasets toward identifying gaps, weaknesses, and a neutral reference architecture. A comparative study of IDSs is provided, with a review of anomaly-based IDSs on DL approaches, which include supervised, unsupervised, and hybrid methods. All techniques in these three categories have essentially been used in IoT environments. To date, only a few have been used in the anomaly-based IDS for IoT. For each of these anomaly-based IDSs, the implementation of the four categories of feature(s) extraction, classification, prediction, and regression were evaluated. We studied important performance metrics and benchmark detection rates, including the requisite efficiency of the various methods. Four machine learning algorithms were evaluated for classification purposes: Logistic Regression (LR), Support Vector Machine (SVM), Decision Tree (DT), and an Artificial Neural Network (ANN). Therefore, we compared each via the Receiver Operating Characteristic (ROC) curve. The study model exhibits promising outcomes for all classes of attacks. The scope of our analysis examines attacks targeting the IoT ecosystem using empirically based, simulation-generated datasets (namely the Bot-IoT and the IoTID20 datasets).

## 1. Introduction

IoT technologies communicate without the need for human-to-human or human-to-computer interaction. IoT has increasingly been adopted by organizations to streamline their operations and is one of the fastest growing technology fields; by the end of 2030, estimates have IoT at 50 billion devices, which includes everything from smartphones to kitchen appliances [1]. IoT innovations are contributing to improvements across real-life smart applications (e.g., cities, healthcare, transportation, and education). Concomitant cutting-edge and large-scale adoption of IoT technology has introduced new security challenges. Adherence to IoT security requirements is hindered by the complexity and integrative arrangements of new and somewhat ad-hoc contexts. IoT devices are connected mostly over wireless networks and are typically utilized in an unattended fashion. In this type of environment, an attacker may easily gain both physical or logical access to these devices illegally. An attacker with assumed malicious intent may indeed cause critical, life-threatening consequences.

To counter the IoT security conundrum, researchers first opted for adopting conventional security mechanisms, including encryption, authentication, access control, network security, and application security. However, such adoptions of security technologies have proved inadequate and have needed enhancement to suit the various contextual needs of their respective environments. Nevertheless, implementing security measures against specific security threats has usually been effective, though often thwarted by new attack Methods and Tactics (M&T). For example, the Mirai botnet caused large-scale Distributed Denial of Service (DDoS) attacks by exploiting IoT devices. While amplifying DDoS, these recent attacks utilize spoofed-source IP addresses to circumvent current solutions targeted to the Mirai botnet M&T. These solutions have motivated newer, more sophisticated attacks that are more complex and more destructive than the original Mirai botnet attributed attacks. Therefore, investigating effective IoT security countermeasures remains a research priority.

IDSs are one promising avenue for monitoring IoT environments and are mainly effective at the network level. IDSs deployed in IoT environments analyze network data packets and generate real-time responses. To be effective, these IDSs need to operate under stringent IoT conditions of low energy, low process capacity, fast response, and notably huge volumes of data processing. Thus, enhancing IoT embedded IDSs is a continuous and serious issue requiring a significant understanding of the security vulnerabilities of IoT systems.

### 1.1. Noteworthy Survey and Key Aspects

Many related surveys on IoT already exist in the literature that cover different aspects of deep learning in cybersecurity. Our comparison of previous studies is based on several key properties as shown in Table 1. These surveys [2,3,4,5,6,7,8,9,10,11,12,13] provide a modest focus on IoT intrusion detection. Most studies are either descriptive of the IoT architecture, or they present the various IDSs as a general overview for a particular project evaluation and verification purpose. References [2,3,6,11] are completely dedicated to IoT architectures and include an incomplete assessment of some applications and protocols. References [4,12] propose a six-layer architecture for the IoT domains. However. IoT security and IDSs were not considered in their study. In [13], the architecture, protocols, and privacy are described only as brief IoT security concepts, including the interconnection between the objects of things. In [7], the authors presented a survey of IDS in IoT but nothing about DL/ML techniques in IDS. Several attacks targeting protocol topology (the Routing Protocol for Low-Power and Lossy Networks (RPL), IPv6 over Low-Power Wireless Personal Area Networks (6LoWPAN)) are discussed in [5] without classifying those attacks on an IoT layered architecture connected with IDS. Reference [8] similarly provides a classical comparative analysis for several existing papers based on advantages and disadvantages. Their focus, furthermore, concentrates on the attacks without due consideration of the ML/DL methods as a general solution.

A few studies provided an extensive background on all IoT areas through an enhanced IoT security (based on IoT-specific threats) approach [6,11,12,14,15]. However, they did not examine, as we have, all of the domains of anomaly-based-IDS for IoT. Table 1 describes those studies that show the preeminent role of anomaly-based IDS for the security of things. This table identifies the gaps in the previous survey studies within the standard architecture layers of IoT systems and then links them with IDSs, such as anomaly-based IDS, with the intent to clarify their solution context and mechanism (An anomaly-based intrusion-detection system is an intrusion detection system for detecting both network and computer intrusions and misuse by monitoring system activity and classifying it as either normal or anomalous).

### 1.2. Contribution and Paper Organization

Accordingly, the thesis of this paper is as follows:

1. IoT architecture standards in term of compatibility and difference between those standards are discussed. This reconciles and creates a mapping between those various IoT architectures with respect to IoT security aspects making the IoT ecosystem robust against intrusions.
2. A novel comprehensive taxonomy is presented that includes state-of-the-art deep learning for IoT-IDS in terms of (a) IoT targeted attacks, (b) IoT architecture, (c) various IDSs, (d) deep learning approaches, and (e) common IoTIDS datasets. The potential attacks and requisite security needs are proposed for each IoT layer defined in Table 1.
3. A fine-grained review on anomaly-based IDSs in the IoT ecosystem using deep learning approaches and traditional anomaly-based IDS approaches is provided. A comparative and descriptive analysis of different anomaly-based IDS approaches in terms of strategy, advantage, and disadvantage is also presented.
4. An experimental study of the performance of four ML approaches, (a) LR, (b) SVM, (c) DT, and (d) ANN, is performed using the Bot-IoT [16] and IoTID20 datasets [17].

Going forward, this article is organized as follows: The taxonomy of deep learning for IoT-IDS security is discussed in Section 2. Security issues and challenges associated with IoT systems are presented in Section 3. Section 4 discusses IoT standards and paradigms. Section 5 examines existing IDS systems used in the IoT environment, including their different detection techniques. Experimental examples, results, and a discussion are found in Section 6. The experiments described in Section 6 were conducted to ascertain and validate the expectation that, within the context (i.e., diverse IoT (i.e., as defined by the data sets)), preliminary proof-of-concept compositions (i.e., ML Models and flow charts) had (1) never been tried before and (2) were able to (preliminarily) perform better than expected and thus (3) are the basis for pursuing more extensive experimentation to establish a more empirical explanation. Section 7 presents future directions and conclusions.

This paper is oriented towards illuminating and surveying the existing state-of-the-art technologies in IoT IDSs and evaluating them based on what various methods can/cannot accomplish. Therefore, we propose an ML and DL framework by which the researcher should abide to build a correct ML/DL model. Moreover, we introduce how to build deep learning-based IDS architecture as a framework in Figure 4. In addition, in Figure 5, we present our model depending on the process of building the model, described in Figure 4.

## 2. Taxonomy of Deep Learning for IoT-IDS Logic

**Figure 1:**
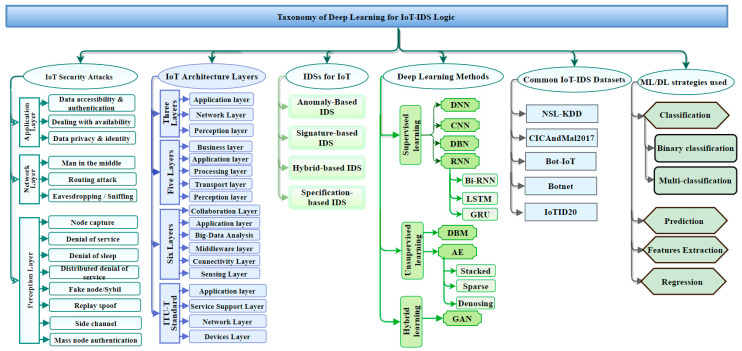
Taxonomy of Deep Learning for IoT-IDS Logic.

Hindy et al. [15] classified various common threats using the seven-layer OSI model. Those various threats are presented as a taxonomy here based on the tools need to carry out said attacks. In [18], the authors presented an overall taxonomy based on public IDS-established datasets. The references [3,4,11,12] provided new IoT architectures and classified current IoT architecture. Other investigators have focused on deep learning techniques, which are classified deep learning methods based on their view of knowledges. In [19], for example, the authors reviewed deep learning-based IDS taxonomy, whereas in [20], the authors provided a taxonomy based on machine learning methods. This section classifies deep learning for IoT-IDS through various aspects. The taxonomy described in Figure 1 houses the aspects associated with IDS expertise by facilitating industry, government, and investigators to develop an intelligent intrusion-detection system in the IoT ecosystem. Figure 1 provides a detailed taxonomy of deep learning approaches used in IDSs. The taxonomy includes the various areas that are important to understanding IoT security issues and their solutions. The taxonomy includes (1) IoT security attacks, (2) IoT architecture layers, (3) intrusion-detection systems for IoT, (3) DL techniques used in the IoT IDSs, (4) common datasets used in the evaluation of the DL systems, and (5) their classification strategies. The different areas included in the taxonomy are in various ways interconnected as root causes of IoT security vulnerabilities in IoT and/or solutions to counter such causes.

### 2.1. IoT-Targeted Attacks

**Figure 2:**
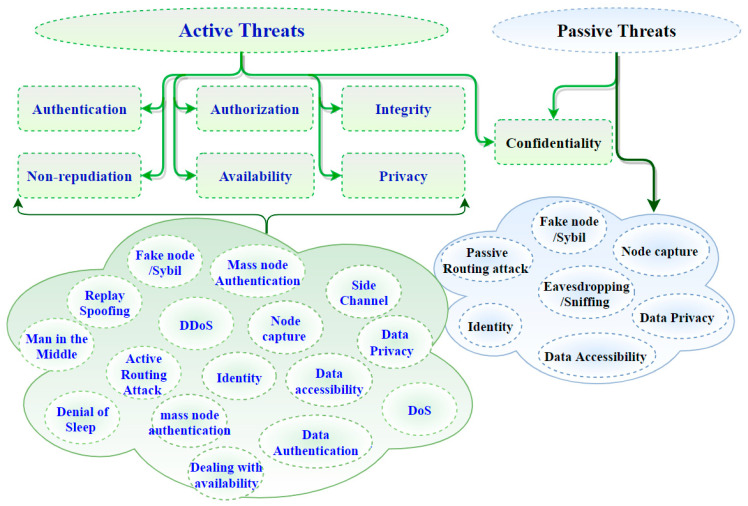
Passive and active threats in the IoT system.

In Figure 1, on the leftmost branch, IoT security attacks are enumerated along with the corresponding layer needed to detect them. Indeed, IoT architectures are vulnerable to various threat actors and attack methodologies. These attacks could be passive or active and internal or remote, as seen in Table 1 and Figure 2. The passive attacks monitor for vulnerabilities and do not disturb IoT ecosystem services (i.e., collecting information needed for future penetration attempts). Active attacks disrupt (i.e., interrupt/block) the operation of targeted IoT devices or IoT ecosystems. These attacks and threats include but are not limited to the methods listed in Figure 1 (e.g., data accessibility, man-in-the-middle, denial of service, distributed denial-of-service attack, eavesdropping, sniffing, routing attack, sybil, replay spoofing, and mass node authentication). Section 3 explains more about the challenges of IoT security Issues.

### 2.2. IoT Architecture Layer

**Figure 3:**
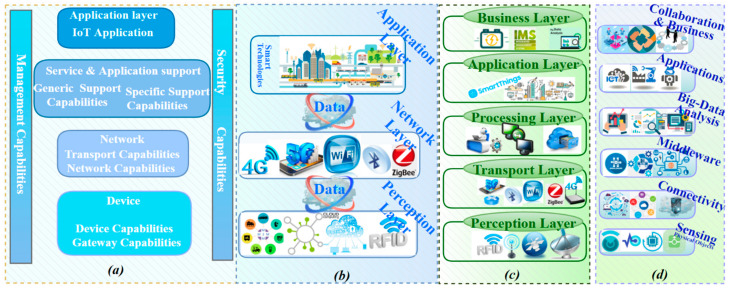
Comparison and layers mapping of (**a**) ITU-T functional architecture view [54]; (**b**) three-layer IoT architecture; (**c**) five-layer IoT architecture; (**d**) six-layer IoT architecture.

Different architectures (see layers 3, 4, 5, and 6 in Figure 1) have been identified by various vendors and researchers. Section 4 discusses and compares the most popular architectures. Section 4.3 proposes a mapping between the literature architectures to facilitate the understanding of the layers proposed by entities and academia. Figure 3 summarizes the layers in the proposed taxonomy.

### 2.3. Intrusion Detection System (IDSs)

Many studies have proposed, developed, and empirically evaluated different approaches for IDSs [21,22,23,24,25,26]. There are primarily four different categories as shown in Figure 1: (1) anomaly-based intrusion-detection system (AD-IDS), (2) signature-based intrusion-detection system (S-IDS), (3) hybrid-based intrusion-detection system (Hybrid-IDS), and (4) specification-based IDS. AD-IDS depends on established known patterns for normal behavior. Behavior outside the realm of “normal” is considered anomalous, thus causing some sort of warning or alert. S-IDS relates to the known pattern (signature) of malicious traffic to detect attacks. The zero-day (unknown; never been seen before) attack cannot be detected by S-IDSs. Specification-based IDS and hybrid-IDS attempt to leverage complementary capabilities by integrating the first two types (AD-IDS and S-IDS). ML and DL algorithms are good examples of the core capability used in AD-IDS. The snort tool is an excellent example of S-IDS [27,28,29]. Other important considerations and details related to IDSs are examined in Section 5.

### 2.4. Deep Learning (DL) Approaches

DL algorithms can be organized into three different types, as shown in Figure 1, based on their functionality and structure: (1) supervised, (2) unsupervised, and (3) hybrid (semi-supervised) algorithms. The common supervised, unsupervised, and hybrid algorithms are all used to protect IoT systems. Supervised algorithms include Deep Neural Network (DNN) [30], Convolutional Neural Network (CNN) [31], Deep Belief Network (DBN) [32], Recurrent Neural Networks (RNN) [33], Bi-Directional RNN (Bi-RNN) [34], Long-Short-Term Memory (LSTM) [35], and Gated Recurrent Neuro Networks (GRU) [36]. Unsupervised algorithms comprise Deep Restricted Boltzmann Machine (DBM) [37] and Autoencoder Neural Network (AE) [38]. The Generative Adversarial Network (GAN) is an example of the hybrid approach. DL Algorithms contain a sequence of many common hidden layers. Artificial neural networks (ANNs), usually simply called neural networks (NNs), are computing systems vaguely inspired by the biological neural networks that constitute animal brains. ANN is considered the simplest neural network. DNN is considered the most complicated neural network due to multiple “hidden” layers. DNNs are widely used in various applications, such as network security, image recognition, and speech-recognition systems. All methods can be used for binary classification, multi-classification, and prediction as well as relatively high feature extraction to enable data reduction and faster convergence times [39,40]. To improve accuracy and to obtain a low false-negative rate, one must employ feature engineering. This considers techniques, such as converting non-numeric to numeric, normalization, and scaling, during the deep learning model development phase. Section 5.3 deeply examines recent studies and findings for the DL class of algorithms.

### 2.5. Common IoT Datasets

As shown in Figure 1, the list of datasets used to validate DL approaches and specifically IoT cybersecurity are substantial. The number and diversity of studies and citations substantially contributed to establishing the structure and criteria of the taxonomy. The datasets that were selected include: (1) NSL-KDD, (2) CICandMal2017, (3) Bot-IoT, (4) Botnet, and (5) IoTID20. All selected datasets are publicly available. Section 5.4 explains in greater detail the nature of each.

### 2.6. Deep Learning Strategy

DL models can be categorized based on the primary goal for the analysis, such as classification, feature extraction, prediction, and expression. The feature-extraction technique plays a significant role in extracting important features, especially in high-dimensional data, such as IoT ecosystem. Feature extraction is significant for creating a suitable prediction or classification model. Most studies describe how to create non-handcrafted features of the data as the basis for training their IDS model for the purpose of enhancing the quality of classification, prediction, and/or regression outcomes. In classification, the model organizes the existing traffic data into two classes, benign (normal) or malicious traffic (a binary classification), with the goal of minimizing false-negative and false-positive rates. Another strategy is to create a model that can handle multi-classification to categorize the abnormal patterns into different malicious attack types. To build a robust prediction model, the feature extractions must be carried out before building the predictive application. A prediction model analyzes the past data and generates a predictive model to forecast future data. It may be a possible solution for transmission issues of IoT sensors data to cloud applications. A prediction model plays an important role to solve spatial-temporal problems in IoT ecosystem. It plays an important role in improving industrial IoT products, reducing the cost, and providing good decision making. The regression model comes with two different kinds of regression: linear regression and nonlinear regression. It fits the time-series problems. It began to surface in IoT ecosystem as one of the solutions for spatial-temporal problems, but it remains the least popular in the IoT research community. To preview those strategies, refer to Figure 1.

## 3. IoT Security Challenges

One important challenge reported in the literature [2,3,4,5,7,8,9,10,11,12,13] is securing IoT technologies, which can be life threatening. A harmful scenario can result with Integrated Smart-Devices (ISD) when exploited by hackers, especially in industrial IoT applications or Internet of Vehicles (IoV). There is a number of IoT technology-hacking scenarios as illustrated in [41,42] that could cause a high level of harm to the system. IoT information security issues are associated with the preservation of authentication, authorization, integrity, confidentiality, non-repudiation, availability, and privacy [43,44]. Security issues and challenges related to IoT technologies can be approached from aspects of issues associated with different IoT layers. Some studies [4,6,45] have proposed security requirements for each layer within the IoT architecture separately, whereas some other references [8,12,25,27,28,29] remain focused on analysis and presentation of the potential threats that attack each layer. This paper seeks to combine security requirements against threats to propose a three-layer IoT architecture. Accordingly, the most basic IoT architecture, the three-layered architecture, provides a simple platform from which to present security requirements and concerns as well as threats/exploits at each layer of the architecture as illustrated by considering Table 2 combined with Figure 2.

The security requirements of Table 2 are defined here. Authentication is confirming the identity of a claimer. Thus, in IoT, each device is expected to have the ability to verify the identity of its user and another device for the interaction with others. Authorization is giving access to an entity to interact in the IoT environment. Integrity refers to maintaining the consistency, precision, and dependability of information, while confidentiality is about making sure that sensitive information is accessed by authorized entities. Non-repudiation guarantees holding an entity accountable for its actions. Availability ensures that IoT services are there and can be accessed from anywhere and anytime the user needs them. Privacy is a property and/or process of ensuring that private information is only accessible by authorized entities. The properties above, taken as requirements, should be enforced to achieve the highest levels of safety. However, IoT device constraints will naturally limit the extent and depth achievable, which therefore necessitates a risk assessment to understand better the threats, impacts, and tradeoffs. Figure 2 shows how active and/or passive threats can impact those aforementioned properties within the IoT ecosystem [12,36,37,38].

## 4. Standards, Paradigm Uptake and Inherent Vulnerabilities

The IoT technology sector comprises different technologies within various application domains. A plethora of terminology has emerged and is used by different vendors to refer to many sorts of IoT domains (i.e., application scenarios and configurations) with vastly different degrees of criticality. Devices with the same purpose, similar functionalities, and structure can be connected into the various circumstances that often represent investments justified by a smarter, more sustainable infrastructure. The critical technologies underpinning IoT include Machine-to-Machine communication (M2M), Internet of Vehicles (IoV), Internet of Energy (IoE), and Internet of Sensors (IoS).

In the history of IoT, the technology has seen tremendous uptake and adoption. IoT-based applications produce huge volumes of data that represent billions of objects communicating amongst each other. IoT is often tightly integrated with cloud computing from fog-nodes through various stages of processing and storage. This layered structure aids in the flow of data between the three layers, where data is autogenerated from an IoT sensor and stored automatically in the cloud [46]. Unfortunately, existing proposed frameworks for the data compression process lack a data-protection scheme to protect or avoid data spoofing/exfiltration and/or integrity at various points within those layers. The very nature of the IoT charge and its vast and ambitious domain of application give rise to inherent vulnerabilities, which have been a driving force in choosing vendors, configurations, and protocols. There have been numerous but mostly proprietary reference architectures thrust into the marketplace.

### 4.1. IoT Reference Architecture

There are several different IoT architectures proposed by vendors, such as the examples by Microsoft [47], SAP [48], and Intel [49], which are shown together in Figure 1. These are a subset, excluding Cisco, Boeing, and others. There are several other architectures proposed by researchers, though there is no one internationally agreed upon IoT reference architecture [50]. However, numerous commercial interests promote their own proprietary structures as being open. A generalized IoT architecture proposed in the literature should consist of three basic layers: perception, network, and fog/cloud (i.e., application) layers as shown in Figure 3b [2,51,52,53]. The layers include the perception layer, which is the physical layer that senses the environment to observe and measure the physical properties using smart devices that employ different sensing technologies. The network layer oversees/controls the receiving of data from the device layer and the transmitting of data up to the application layer via various network protocols. Finally, the application layer provides application-specific services to consumers (e.g., Losant Enterprise IoT Platform, Smart City, Intelligent transportation system, Hewlett Packard Enterprise, IBM, etc.).

Another popular IoT architecture proposed in [45,54,55] consists of five layers as seen in Figure 3c: perception (i.e., physical), transport (i.e., network), processing (i.e., middleware), application, and business layers. The supplementary layers are the processing layer and the business layer. The processing layer is a middleware layer responsible for providing various types of services, including storage, analysis, and post-processing of data. The business layer is the overarching “overall” IoT system where big data analytics are conducted, and the decision-making process regarding business strategies and roadmaps is conducted.

Reference [12] proposes a six-layer architecture: physical objects, connectivity, middleware, big data analytics, applications, and collaboration and business objectives layers as shown in Figure 3d. The physical objects layer (i.e., sensing) comprises sensors and actuators of the IoT ecosystem. The plug-and-play (PnP) configuration phase of IoT-devices happens at this layer, providing interconnection within a heterogeneous environment. A key step for fulfilling a context-aware IoT ecosystem is understanding the sensor data supplied by those devices. Therefore, the connectivity layer is a key target of malicious attacks due to its typical ad-hoc nature. The connectivity layer typically includes collaboratively connected heterogeneous sensors in designs that seek to achieve cooperative fault-tolerant goals. The objective of a middleware layer is to provide a versatile interoperability layer that enables developers to concentrate on solving the problem without interruption at the level of the software and/or hardware within the ecosystem. A big-data analytics layer builds in IoT intelligence by providing smart services. This layer can leverage ML/DL to play an analytical role using data captured within the ecosystem. The application layer consists of several smart applications, such as smart transportation, smart agriculture, smart robot, smart healthcare, etc. Finally, the collaboration and business objective layer is used to enhance and improve a multitude of applications from smart living to numerous commercial industrial types through efficient use of data collection, distribution, and evaluation at different levels of the ecosystem. Similarly, the authors in [4] consider six layers as their adopted architecture but use different nomenclature. The layers begin with sensing at the bottom, short-communication, gateway access, network, service platform and enabler, and application at the top of the stack.

### 4.2. IoT Standards

Several criteria (i.e., standards) to baseline IoT architectures have been proposed by standards organizations, such as ITU Telecommunication Standardization (ITU-T) [56], and related open-standards organizations (e.g., advancing network functions virtualization (NFV) and software-defined networking (SDN)). Intel’s involvement in these open-standards organizations includes 3GPP, Cloud Native Computing Foundation, European Telecommunications Standards Institute, Linux Foundation Edge, Open Network Foundation (ONF), Open RAN Alliance (O-RAN Alliance), Internet Engineering Task Force (IETF), and Open Container Initiative (OCI) [57]. Such standard initiatives aim at facilitating interoperability, simplifying development, easing implementation, and identifying both functional and non-functionable weaknesses in the IoT systems. Figure 3a illustrates the functional view of the IoT standard proposed by ITU-T [56]. The architecture consists of four layers: the (1) device, (2) network, (3) service and application support, and (4) application layers.

### 4.3. Architecture Mapping

In this section, a comparison mapping between various proposed architectures with the ITU-T standard as illustrated in Figure 3 is developed. Mapping the layers from Figure 3c to Figure 3b, the business, application, processing, transport, and perception layers correspond functionally to the three layers (perception, network, application) in the latter architecture, respectively. The network layer in Figure 3b could be split into two different layers (processing and transport layers) as shown in Figure 3c. Similarly, the application layer in Figure 3b corresponds to the business layer and the application layer.

While there are myriad pieces in the puzzle that build complete end-to-end IoT architectures including both cutting-edge and legacy technologies as well as broadening applications areas, there is no single architecture considered to be suitable for all areas across the board. Most of the architectures can be considered an extension to the basic IoT model. We conducted our analysis to provide evidence of convergence by matching various architectures with one another. This analysis provided an approach to support affinity matching across architectures and mapped the five-layer architecture and the six-layer architecture as follows: (1) sensing (physical objects) layer in Figure 3d corresponds to the perception Layer in Figure 3c; the (2) connectivity layer coincides with the transport layer; the (3) middleware and big-data layers match the processing layer in Figure 3c; while the (4) application layer match the application layer and the (5) collaboration and business layer in Figure 3d correspond to the business layer in Figure 3c. The logic for the mapping from Figure 3d to Figure 3b is given therefore as follows: the (1) sensing layer in Figure 3d corresponds with the perception layer in Figure 3b; the (2) connectivity, middleware, and big-data analysis layers in Figure 3d correspond to the network layer in Figure 3b; and the (3) application and collaboration and business layers in Figure 3d correspond to the application layer in Figure 3b.

The mapping logic endures within recently proposed architectures. The ITU-T standard with the six-layers architectures is roughly similar when overlapping between layers is considered. ITU-T standard as shown in Figure 3a consists of four layers, which can be mapped with the six-layer architecture in Figure 3d by the following representation: the device layer corresponds to the sensing layer, the network layer comprises two functionalities distributed among two different layers (connectivity and middleware layers), the service and application support layer corresponds to big-data analysis layer, and the application layer coincides to both application and collaboration and business layers in Figure 3d. The compatibility between the ITU-T standard and the basic architecture of the three layers and five layers can be deduced through the following compatibility: the device layer in Figure 3a corresponds to the perception layer provided in Figure 3b,c, the network layer corresponds to the network layer in Figure 3b and the processing layer and transport layer in Figure 3c, the service and application support layer and the application layer in Figure 3a correspond to the application layer in Figure 3b (with a difference to the application layer in Figure 3b cannot provide full service and support as same as service and application support in Figure 3a), and the application layer in Figure 3a corresponds to the application layer in Figure 3c, whereas the service and application support layer in Figure 3a corresponds to the business layer in Figure 3c.

## 5. Intrusion Detection System (IDS) in IoT

Security practitioners use monitoring systems to discover security vulnerabilities and anomalous, possibly malicious, activities. These monitoring systems use passive traffic collection and analysis to accomplish their objectives. An IDS is a monitoring tool that observes data in network traffic to identify and protect against intrusions that threaten the security of information systems [7,58]. IDSs are best known as the second line of network defense. This security component comes into two forms: (1) host-based (HIDS) and (2) network-based (NIDS). HIDSs monitor activities on the server, whereas NIDS systems monitor network activities and communications. Since normal and malicious behaviors are assumed distinct, IDSs can monitor behaviors of host and network activities for signs of attack [7]. IDS architectures can be categorized into centralized, distributed, and hierarchical. Centralized IDSs monitor data from a central location which, in most cases, is in a remote or host-based location. Distributed IDSs are positioned among multiple nodes within a network with “equally” shared responsibilities. In the HIDSs situation, an IDS node can exist alone or in combination with other types of architectures with evenly distributed responsibilities [6]. IDSs can be misuse-based or anomaly-based. Misuse-based methods use a database of known signatures and patterns to detect well-known attacks as contrasted to anomaly-based systems, where a normal data pattern (i.e., profile) is created based on data from user’s established normal behavior and then compared against current data patterns in an online manner to detect anomalies [7]. Our current research focuses on anomaly-based IDS systems with reference to the systems identified in Figure 1.

Both types of IDSs use different algorithms for detection. Most lightweight IDSs in the literature are favored for use in the IoT ecosystem. These lightweight IDSs systems use a principal component analysis (PCA), which is a lightweight algorithm that employs various detection techniques in IDSs [59]. Consequently, this paper focuses on discussions related to PCA used in IoT anomaly-based IDSs. In [60], the researchers proposed PCA to create an anomaly-based statistical and data-mining IDS that depends on the division of the principal components into the most and least significant principal components. PCA used for intrusion detection is based on payload modeling in [61], statistical modeling in [62], machine learning in [63], and data mining in [64]. Table 3 shows the advantages and disadvantages of anomaly-based IDS approaches employed in IoT as related to the detection modeling techniques [7,65]. Machine learning (ML) algorithms are applied via two stages: the training stage that uses mathematical algorithms fed with ordinary data to learn the characteristics of the computing environment, followed by a detection stage, where non-ordinary data are used to validate detection and classification [66]. The preferred use of data-mining techniques are for online environments with unbounded, continuous, and rapidly increasing volumes of data to automatically generate models that depend on the traffic description [67,68]. The Payload Model processes a packet byte-by-byte in a streaming context from network traffic. This model distinguishes the normal (ordinary) characteristics of network packet traffic on a specific port or for a specific user for a given application from abnormal characteristics to identify attacks [69]. The statistical model uses a kind of stochastic filter operation, such as the statistics of historical user behavior to create a normal profile. Consequently, any deviations from the established norm are then considered abnormal and detected as an attack [70], though not necessarily malicious.

### 5.1. Anomaly IDS-Based Traditional Detection Approaches in IoT

In IoT environments, anomaly-based IDSs are used to monitor the behavior of a normal network and to define a threshold to detect deviations from the normal behavior [71]. In this section, we review existing anomaly-based IDSs proposed for the purpose of protecting the security of IoT environments. We study different detection techniques employed in each of the reviewed systems. For example, in [72], the researchers present an anomaly-based IDS system that uses data-mining techniques as a distributed intrusion-detection scheme to detect anomalies in IoT environments. Their research theoretically showed, by using the intrusion semantic to distinguish intrusive from a normal behavior, that the proposed approach is accurate and extensible. Ding et al. [73] proposed a non-cooperative differential game model that uses statistical techniques to allow all nodes in an IoT environment to choose the optimal amount of network resources to invest in information security contingent upon the state of the game. This research models selfish-nodes and malicious-nodes interactions as a differential game. The results show that malicious behavior can be discovered with high probability and high detection accuracy, good performance, and low resource consumption. Chen et al. [74] proposed a fusion-based approach for attack inference at the IoT network level. The approach details the attack and IDS procedure as a zero-sum game. The outcome of the game equilibrium is used to evaluate the network robustness achievable from a given proposed defense mechanism.

Rajasegarar et al. [75] proposed a distributed anomaly-based IDS approach that utilizes numerous hyper-ellipsoidal groups to show the information at every node and detect global and neighborhood abnormal behavior within the system. The approach uses a novel scoring-based technique that provides a score for each hyper-ellipsoidal model, achieving a higher detection performance. The approach is proposed for resource constrained networks, which makes it suitable for use in IoT environments. Ham et al. [76] proposed a machine learning-based IDS approach that employs the SVM to distinguish anomalies on android for the IoT services. The approach uses behavioral-based detection to enable automatic anomaly classification to ensure detection accuracy. Wong et al. [77] proposed an online anomaly detection method for IoT environments that uses an integrated probabilistic model. The MATLAB simulation environment was used for the implementation and evaluation of this approach. Pongle and Chavan [78] proposed an IDS that recognizes wormhole attacks for IoT. This approach uses a data-mining model to recognize an intrusion by employing a local node with its neighbor node data to identify intrusion by adding a flag inside a victim packet to distinguish malicious nodes. Summerville et al. [61] proposed an ultra-lightweight deep packet anomaly-detection scheme for resource-constrained IoT devices to identify normal and abnormal behavior. their approach uses payload modeling that uses bit-pattern and n-gram sequences. The proposed approach can be implemented on an IoT device or can be built into network appliances and firewalls. Additionally, in [79], the research provided a lightweight IDS that uses payload modeling to detect distributed denial of service (DDoS) over IoT networks. There are a number of other anomaly-based IDS approaches that use previously mentioned detection modeling approaches for IoT environments, including the one proposed in [80] that uses taint analysis to detect attacks on IPv6 within the IoT routing service. Rahman et al. [81] presented a neuro-fuzzy based IDS that identifies incidents at the physical or medium access control (MAC) layer of the IoT. In our previous work [82], we addressed anomaly problems by offering an improved Adaptive Anomaly Detection (AAD) methodology that resolves the heterogeneity issues by building local profiles that define normal behavior at each IoT node.

One of the main shortcomings associated with the reviewed anomaly-detection approaches is the lack of high accuracy specifically when used in IoT. With the ever-increasing complexity of attacks, the traditional detection algorithms, such as classical machine learning, statistics, payload, and mining-modeling techniques, are incapable of detecting complex cyber breaches [83]. As a result, researchers have opted to use deep learning in intrusion-detection systems and have shown that it could have a novel application in anomaly detection for IoT [84,85,86]. Deep learning has been improved over ML in many computing domains due to current developments in hardware and powerful deep learning algorithms (Deep learning (DL) algorithms, a subset of machine learning, are characterized by their complexity (or depth) of the neural network (NN) hidden layers. ML contains either linear or nonlinear algorithms as a single layer. In ML, the feature extraction (selection) is the first step that precedes the implementation of the model, while in DL, the feature extraction is embedded within the model). The huge volumes of data generated in cutting-edge technologies also make a tremendous contribution to the current adoption of deep learning in intrusion detection.

### 5.2. Deep Learning (DL) Approaches

DL has been applied to a myriad of problems. Here, we highlight recent advances from the perspective shown in Figure 1, namely protecting IoT security. DL is a state-of-the-art feature extraction and classification method well known within the domains of image recognition and data processing. It extracts huge, complex, and nonlinear hierarchical features to build models that transform inputs to an output, so-called as ANN algorithms, with more than two layers of neural network [87]. Numerous different DL algorithms exist. The preferred use of DL is feature extraction and classification [88]. DL uses interconnected neurons to jointly perform a non-linear transformation of inputs to certain desired outputs. In IoT security, DL architectures are a powerful method of data exploration to learn about normal and abnormal behaviors. DL techniques are used in IoT security because they can perceptively predict future unknown attacks. Data collected, for example, from each layer of the IoT architecture is used as input to determine the normal patterns of interaction, resulting in identification of malicious behavior at an early stage. DL techniques are also used to predict new attacks, which are, in most cases, mutations of known attacks. DL methods are principally used in IoT systems that produce huge volumes of data and are filtered using one of the three supervised, unsupervised, or semi-supervised learning types [89]. The common supervised DL approaches used for the security of IoT systems include DNN [30], DBN [32], CNN [31], RNN [33], LSTM [35],(Bi-RNN) [34], and GRU [36].

DNN, a branch of ANN, provides the backbone structure of DL algorithms, which leverage Multilayer Perceptrons (MLP) composing multi-hidden layer architectures. DBN is a series of stacked RBM layers that execute greedy layer-wise training to achieve robust performance [90]. DBN can be utilized for dimensionality reduction. In DBN, the data are represented by the visible layer, while the hidden layer transforms to represent the characteristic features. The technique learns how to perform the processing while training. CNN also allows automatic feature learning and reduces the data parameters compared to conventional machine learning approaches. CNN uses sparse interaction, parameter sharing, and equivariant representations to accomplish data parameter reduction. The CNN approach has two alternating types of layers: convolutional layers and pooling layers. CNN has another essential part called the activation unit, which performs a non-linear activation function called the rectified linear unit (ReLU, i.e., f(x) = max (0, x). This function takes the value (x) from the previous neuron (and zero (1st parameter)), thus making the output of the current neuron passed to the next neuron (i.e., if greater than zero; making all negative values of x set to zero). The training time and scalability of CNN architectures can be improved by reducing the connections between its layers. Several researchers have used CNN for IoT security [91,92]. The RNN approach, on the other hand, handles sequential data and is used for applications that consist of sequential inputs, such as sensor data. IoT devices generate many sequential data from sources such as network traffic flows (e.g., in [93]). Investigators have debated RNN-assisted networks for secure and reliable IoT storage. LSTM was first proposed as an implementation of the RNN [35]. The LSTM architecture is different from RNN; it is trained for cases that require state awareness, as LSTM can retain the knowledge of earlier states. In addition, there is a lightweight version of LSTM derived from GRUs [36]. GRU aims to solve the vanishing gradient problem reported in standard RNN architectures. GRU can be considered as a simpler architectural variation on LSTM, which uses a gating mechanism. GRU is known as a LSTM with a forget gate and fewer parameters. Bi-RNN [34] puts two RNN together to enable both backward and forward information propagation. Accordingly, Bi-RNN runs inputs in two ways, one from past to future and the other one from future to past. Bi-RNN, with two hidden states combined, can preserve information from both the past and the future, different from LSTM, which runs only backwards to preserve information.

Common unsupervised DL approaches include AE and DBM. Deep AE methods are introduced in [38] to produce better data representation resulting from dimensionality reduction. An AE, apart from the hidden layers of low-dimensional features, consists of an equal number of feature vectors for each input and output layer. An AE combines an encoder that extracts features from the entire dataset and learns to convert the input into a low-dimensional representation. The decoder receives the low-dimensional representations and reconstructs the original features [94]. RBM neural network introduced by Ackley et al. [95] contains fewer hidden layers and has been used in many domains, including securing IoT systems [96,97]. RBM, unlike AE, consists of two types of layers: input layers, which are the visible layers, and hidden layers. The main motive behind RBM is to limit the number of features processed by each layer. RBM has limited feature-representation capability and is substantially stacked from two or more RBM layers to form a DBN. In [37], the authors produced a new learning algorithm based on a fully connected Boltzmann machine to enhance the RBM technique, so-called DBM, which is an undirected model, as is RBM. DBM, unlike RBM, consists of several hidden layers, whereas RBM only contains one or possibly two hidden layers. Unfortunately, RBM possesses drawbacks that are inapplicable to onboard devices with limited resources. However, investigators still employ RBM/DBM in securing IoT environments. Nevertheless, IoT architectures will perhaps be adapted to accommodate the use of DBM, thereby streamlining the use of ANN countermeasures.

Hybrid or semi-supervised DL methods combine generative features in early phases and discriminative features at a later stage for data differentiation. Generative adversarial network (GAN) is a good example of hybrid deep learning. GAN has been adapted into the IoT environment for security purposes [98,99]. GAN may show improved success because it can learn different attack scenarios that are combined to generate samples similar to a zero-day attack scenario. Such predictive capabilities represent a higher level of learning and require that such hybrid algorithms receive extra attacks samples to learn other than existing attacks [12] that approximate suspicious zero-day behaviors. This course aims to achieve lower false-negative rates, though perhaps at the expense of higher false-positive rates. Yet, some would argue that higher learning layers are necessary to anticipate unknown, sophisticated attack strategies.

### 5.3. Deep Learning-Based IDS Architecture

**Figure 4:**
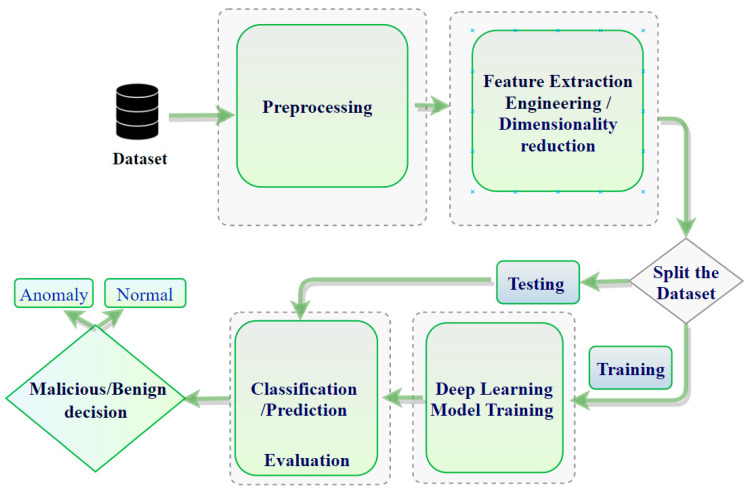
Diagram of the framework of IDS based on deep learning.

Traditional detection techniques, noted previously, have fallen short of detecting new complex attacks. As the volume of data increases, for example, into terabytes, it has become even more important to find alternative techniques. DL models can train using massive amounts of data to build robust anomaly detection systems. The model classifies the new traffic into either a normal or anomaly class [100]. DL techniques learn from hierarchical discriminative features discernable in the data. The fact that anomalous behavior is often not precisely defined poses challenges for conventional techniques; therefore, domain experts have begun to advocate solving the problem using DL techniques [87]. Some anomaly-based IDSs are used in the IoT context by employing deep learning techniques for their insights. The most common deep learning architectures employed for anomaly detection in conventional systems include CNN [31,101], DNN [30], LSTM [84,102,103], and RNN [104]. Such deep learning architectures are employed in an anomaly-detection system for either feature learning or classification [69]. Figure 4 shows the (typical) overall framework of IDS based on deep learning.

A few studies have used DL architectures in anomaly-based IDSs in IoT. In [105], researchers proposed a distributed anomaly-detection-based IDS for IoT environments. The approach uses Autoencoder Neural Networks (AE), which is a two-part algorithm that resides on sensors and the IoT cloud/Fog, respectively. The anomalies can be detected at sensors in a distributed manner, while the computing burden is handled in the cloud with a lower frequency. The researchers use their own dataset to test the performance of the proposed approach. The authors [106] proposed a hybrid spectral clustering-based detection technique using DNN in sensor networks. They utilized the KDD99 and NSL-KDD datasets to detect intrusion behavior. They employed k-clusters for feature extraction from the entire dataset and to evaluate the performance of their model. The authors of [107] utilized LSTM to propose a model to predict the operating state of IoT equipment by analyzing the data collected from IoT sensors. In [108], the authors implemented LSTM on the Coburg Intrusion Detection Datasets (CIDDS) to create an IDS for classification problems. They applied LSTM to generate their model in a simple way. They compared their model (LSTM-IDS) with ML methods (SVM, Random Forest, Naive Bayes, and ANN). They achieved higher accuracy using LSTM-IDS: close to 85% more than other methods. Their model’s weakness is that it classifies only known attacks accurately, with no accounting for zero-day type attacks. Pamukov et al. [109] suggested a classification algorithm that uses DL in anomaly-detection-based IDS. The approach uses a negative selection algorithm for training the system and a simple neural network to conduct the actual classification. The negative selection algorithm creates training data only using normal network behavior data. The approach also uses the R-continuous Bit-Matching rule as a classification function. To evaluate the performance of the approach, they used the NSL-KDD dataset. The model is not an online solution and is not suitable for bringing solutions to areas of large-scale self- or non-self-classification problems.

The study in [110] proposed an ANN to detect DDoS attacks. The neural network is trained on a labeled data set that learns a mapping from input to output that enables the classification of normal and anomalous behavior. The researchers used their own data to train the proposed approach for performance evaluation. Live data are collected from network traffic from a simulated environment without modification. The performance provided in the evolution part of the approach is promising and has shown that more time is required for good efficiency. The study in Lopez et al. [111] proposed an anomaly-based NIDS system that uses an Intrusion-Detection Conditional Variation Auto-Encoder (ID-CVAE) within the IoT context. They claim the approach is less complex and provides better classification results. The approach provides feature reconstruction to recover missing features from incomplete training datasets. They utilized the NSL-KDD dataset to evaluate their model. The study in [83] proposed an anomaly-based IDS approach that uses DL to enable the detection of attacks in a social IoT. The approach is centralized and can be extended to distributed systems. The researchers utilized theoretical analysis to compare the work with other works that employ ML techniques using the NSL-KDD dataset. The approach provided a good detection accuracy and low false-positive rates, although the downside of the approach is that it needs high training time and resources.

Authors of [112] proposed an adaptive model combining an “improved” genetic algorithm (GA) combined with a Deep Belief Network (DBN) for an IoT-ecosystem IDS. The DBN model was divided into two phases: (1) the training stage, which uses multiple RBM layers, each trained separately, and (2) the backpropagation stage neural network, which was set the last layer of the DBN. The study used the NSL-KDD dataset to apply the model to detect attacks and acquired the accuracy from 97.78 to 99.45% for various attacks. The study used GA to adopt an optimal network structure. In [113], use of DBN and DNN and an anomaly-based IDS approach was proposed. The approach is a near real-time detector that provides effective detection. The researchers used the approach in real-network traces to provide proof of concept and simulation for evidence of scalability. The authors in [114] utilized BiRNN with LSTM to propose a DL model to detect malware in IoT ecosystem based on operation codes “OpCodes”. Their dataset was 180 malware and 271 benign files. They acquired a high accuracy (98.18%) in both training and testing data. It is worth noting that there is little research using BiRNN to apply IDS in IoT. In the same context, there are not enough studies using GRU to apply IDS in IoT ecosystem. The authors of [115] applied a GRU to design an effective IDS in IoT ecosystem. Their model was a lightweight IDS for IoT ecosystem in which IDS places at each TCP/IP layer architecture. The KDD 99′cup data set was used by applying their model to classify the data at each IDS device. The DNN technique was used to classify benign network traffic from malicious network traffic, while a perceptual learning model was used for data collection and feature extraction. 

In [60], investigators used the DNN in a distributed attack detection scheme. They did not clearly state the detection method used to achieve the scheme. The approach proposed in [103] used LSTM for outlier detection of attacks in industrial IoT. The method used a predictive error for the Gaussian Naïve Bayes model to classify attacks and was evaluated using three real-life datasets. The results demonstrate promising performance over other methods. Researchers in [102] contributed a host-based anomaly-detection framework that uses Extreme Gradient Boosting (XGBoost) and LSTM. The framework uses abnormalities in the system call sequence as an indicator in real-time experiments to evaluate the performance of both models. The approach uses an N-gram algorithm to extract features and the stacked XGBoost and LSTM for classification. In [101], a framework that monitors abnormality in IoT traffic was proposed. The framework used the Vector CNN approach for classification in Fog environments. The framework was evaluated with the Bot-IoT dataset, and the results provided showed relatively better performance compared to RNN and LSTM. LSTM-based models, previously highlighted, are reported to have better performance than CNN-based models. In [116], a CNN-based network anomaly-detection system using a special layer for packet pre-processing was proposed. The validation used the NSL-KDD dataset. A comparison of the reviewed anomaly-based IDS approaches that use deep learning techniques is provided in Table 4.

### 5.4. IDS Datasets Appropriate for IoT

There are a good number of datasets available for the development and validation of IDSs. The most popular datasets used in the implementation of IoT-IDSs include NSL-KDD [117], the Bot-IoT [16], the Botnet [118], and the Android malware [119] datasets. The NSL-KDD dataset is designed to solve some of the inherent problems of the KDD’99 dataset. Thus, NSL-KDD eliminated redundant duplicate records, thereby significantly reducing the total number of records. The number of borderline (i.e., difficult) records were eliminated based on the inverse percentage so that the NSL-KDD dataset has far fewer borderline records than other datasets. Several papers focused on IoT intrusion detection have used this NSL-KDD and reported judicious and sensible results. The Android malware dataset (CICAndMal2017) contains malware and benign applications, proposed in [119]. The malware samples used to develop this dataset consist of Adware, Ransomware, Scareware, and Short Message Service (SMS) malware and include more than 80 network traffic features. The Bot-IoT is an IoT traffic-based dataset that contains more than 72,000,000 records, including DDoS, DoS, OS and Service Scan, Key-logging, and data exfiltration attacks [16]. The Bot-IoT, compared to other datasets, is dedicated to the validation of IDS within an IoT environment. The Botnet dataset is an internet-connected devices-based dataset containing training and test data that include 7 and 16 types of botnet attacks, respectively [118]. The data featured in the botnet dataset include four groups: *Byte-*, *Packet-*, *Time-*, and *Behavior-based*. Finally, IoTID20 was developed for anomalous activity detection for the IoT ecosystem. It was generated by including laptops, smartphones, Wi-Fi cameras, and other IoT devices. The Bot-IoT and IoTID20 are described in Section 6.2.

## 6. Experimental Results and Discussion

We used two real traffic IoT datasets: the Bot-IoT dataset [16] and the IoTID20 dataset [17]. We selected 5%, which included full features from the Bot-IoT database, while the second dataset was fully selected in the experiment. The proposed model and experiments were trained and tested using the Google Collaboratory (Colbab) with Graphics Processing Unit (GPU), Python, TensorFlow, Scikit-Learn, SkFeature, Numpy, and Pandas.

**Figure 5:**
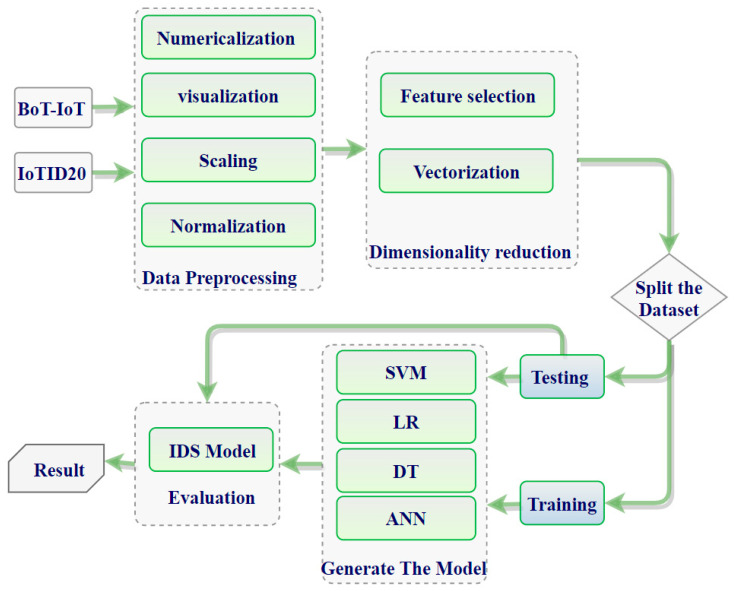
Diagram of our framework of IDS based on the selected ML algorithms.

The process details of the experiments for building the IDS-ML model are shown in Figure 5. The following ML algorithms, namely LR, SVM, DT, and ANN, were employed in this study. The proposed model consists of four stages: (1) data processing, (2) dimensional reduction, (3) training, and (4) testing stage. Visualization preparation and dataset analysis were implemented in stage (1), while ROC requires utilization of all steps to build the model and extract the results.

It is more important to understand the imbalanced dataset because it influences both accuracy and prediction. Effort was devoted to answer the following question: Does ROC-AUC give a better performance rate under various threshold tunings in the unbalanced dataset?

### 6.1. Evaluation Metrics

We used the following well-established metrics to measure model performance. Classification can be further categorized into binary classification and multi-classification. In binary classes, the class labels are either “normal” or “attack.” The outcomes for binary classification must be categorized as follows: (i) true positive (*TP*): when an attack is correctly identified as an attack by the model; (ii) false positive (*FP*): when a benign node is defined as an attack; (iii) true negative (*TN*): when a benign node is correctly identified as non-attack; and (iv) false negative (*FN*): when the model identifies an actual attack as a non-attack (i.e., attackers win and defenders lose). These four categories shape the so-called confusion matrix [120]. To evaluate ML models, the following equations derived from the confusion matrix are (universally) used. These matrices are characterized by the following equations:

(1) $$ACC=\frac{TP+TN}{TP+TN+FP+FN}$$

(2) $$\mathrm{Sensitivity}=\frac{TP}{TP+FN}$$

(3) $$\mathrm{PR}=\frac{FP}{FP+TN}$$

The accuracy (*ACC*) is the percentage of correctly identified cases divided by total cases that were considered (whole populations). Sensitivity is also known as “recall,” or true-positive rate (TPR) or simply the “detection rate.” Sensitivity intuitively indicates the model’s power to correctly identify attacks. Moreover, it provides the ability of the model classifier to find all the positive samples in the training model, while in the receiver operating characteristic curve, or ROC curve plots, FPR is represented on the *X*-axis, and TPR is represented on the *Y*-axis. In other words, the upper-left hand corner of the plot (image) is the “ideal” point, where TPR = 1 and the FPR = 0. Thus, using the ROC curve, efficiency of the model is measured as best when the Area Under the Curve (AUC) is maximized. Accordingly, *ACC*, ROC curve, and AUC (area under the ROC curve) are used to evaluate the model, whereas the ROC curve is a graph used to visualize the performance of classifiers at various threshold settings.

### 6.2. IoT-Datasets

**Figure 6:**
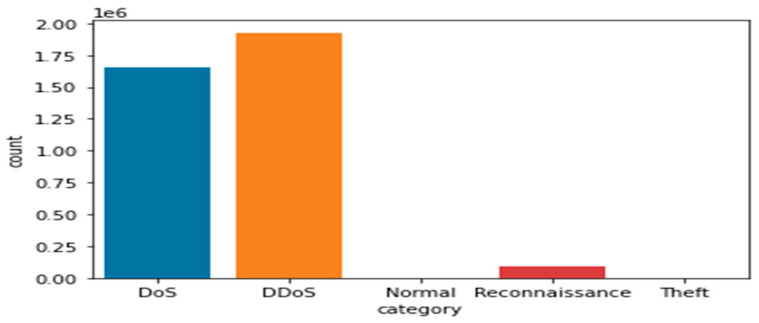
Statistics of considered attacks (the main category) (count is 1 × 1 to the 6th).

**Figure 7:**
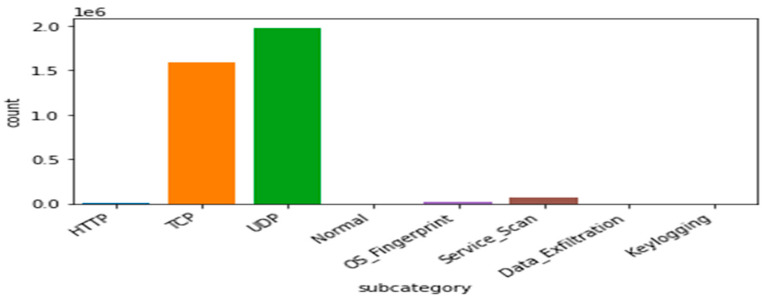
Statistics of considered attacks (the subcategory) (count is 1 × 1 to the 6th).

Bot-IoT dataset: The selection of the BoT-IoT dataset is to display various types of security threats and cyber-attacks. These were categorized into the main category of attacks and the subcategory of attacks as illustrated in Table 5, Figure 6 and Figure 7. Due to the limitations of IoT devices, DDoS and DoS are more prevalent than other attacks. Therefore, the most significant attacks that IoT devices encountered are DDoS and DoS.

Therefore, traditional protection systems, such as Firewalls and Snort, fall short when challenged by such attacks. Table 5 shows a detailed representation of the distribution of various attacks and their anomalies (in just 5% selected data from the whole dataset) and indicates the number of attacks in each category. Table 5 clearly shows that UDP and TCP are the most important services (protocols) affected by malicious attacks. DoS occurs in some unwanted traffic (source and receiver). An attacker sends many vague packets and repeated packets to the victim (target), and as a result, the victim’s services become unavailable to other clients and services. The attacker takes advantage of a weak node to launch an attack through the whole network. Through the vulnerable nodes, the attacker starts sending a large amount of fake data through the network to other nodes.

**Figure 8:**
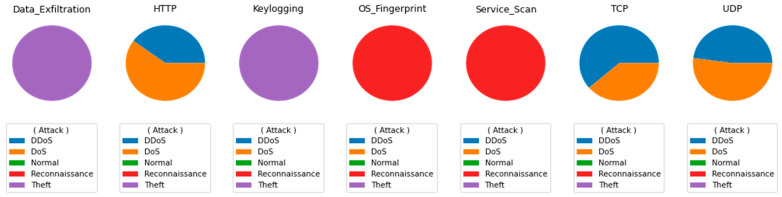
Services Distribution.

Figure 8 shows targeted attack intensity for each of the seven services (from left to right). The service distribution gives a first impression of the scale of challenges confounding the IoT ecosystem. This is a difficult problem that must be addressed and mitigated.

IoTID20 dataset: The IoTID20 dataset was developed for anomalous activity detection for the IoT ecosystem. The data consists of various types of IoT attacks as well as normal traffic (i.e., many features that are present are of a general nature). These attacks include Mirai, DoS, Scan, MITM ARP Spoofing, Scan Host port, and Mirai-UPD flooding.

**Figure 9:**
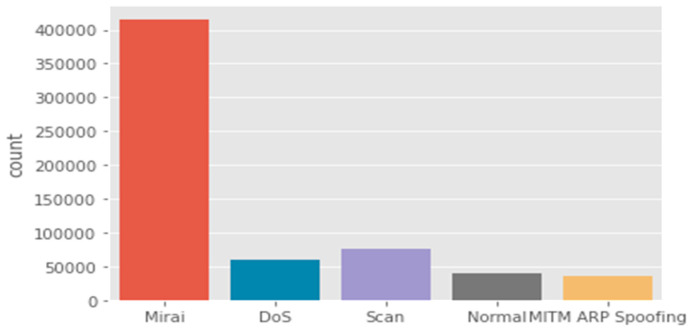
The main categories of attacks are found in the IoTID20 dataset.

**Figure 10:**
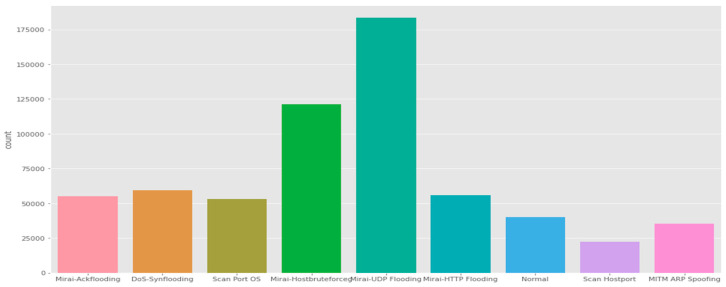
The subcategories of attacks in IoTID20 dataset.

The dataset was generated by including laptops, smartphones, Wi-Fi cameras, and other IoT devices [78]. Effectively, the IoTID20 dataset represents a smart-home environment. The authors derived OpTID20 by splitting the devices within the simulation testbed into two groups. The first group represents the attacking devices, while the second group (e.g., Wi-Fi camera) is the IoT victim devices. The testbed has been implemented to simulate various attacks by using the Network Mapper (Nmap) tool. Figure 9 shows the main categories of attacks that are found in the dataset, whereas the subcategories in this dataset are illustrated in Figure 10.

### 6.3. Result and Discussion

**Figure 11:**
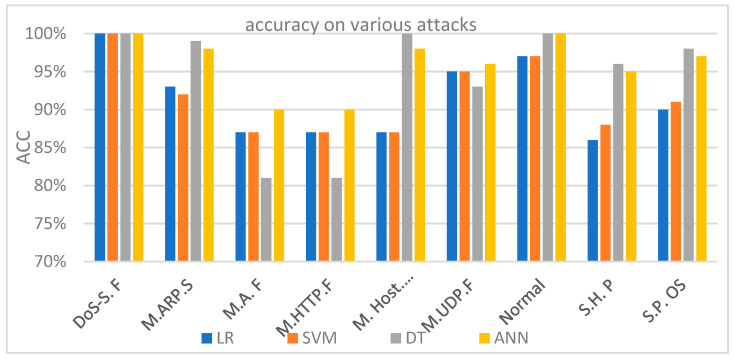
Comparison between ML algorithms.

**Figure 12:**
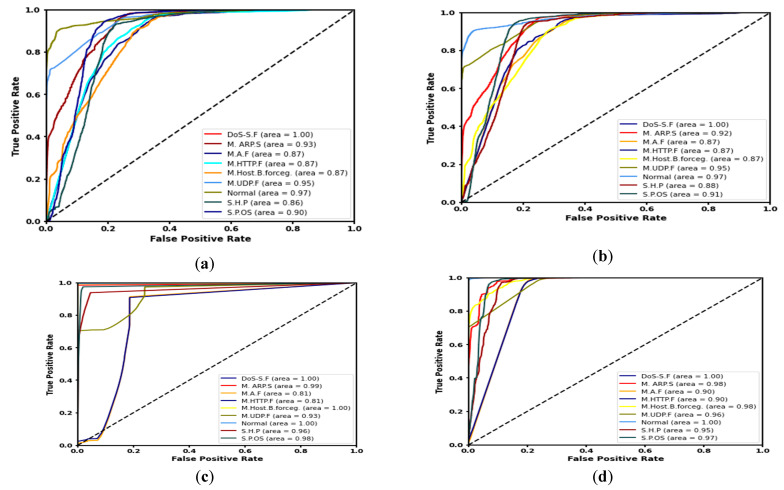
ROC curves for four various ML algorithms (**a**) LR, (**b**) SVM, (**c**) DT, and (**d**) ANN.

Table 6 shows the performance of all four models (LR, SVM, DT, and ANN). Figure 11 shows accuracy for each of the four experiments that were conducted on the IoTID20 dataset. These results indicate the feasibility and efficiency of our approach using the four ML algorithms to detect malicious and normal (benign) nodes. The ROC curves of (1) LR, (2) SVM, (3) DT, and (4) ANN are discussed and shown in Figure 12. The ANN model produced the highest detection rate (DR) for three attack types, namely Mirai-ACK flooding (M.A.F), Mirai-HTTP Flooding (M. HTTP.F), and Mirai-UDP Flooding (M.UDP.F). On the other hand, the DT model produced the highest detection rate (DR) for MITM ARP Spoofing (M.ARP.S), Mirai-Host brute force (M. Host. B. force), Scan Host-port (S.H. P), and Scan Port OS (S.P. OS).

The ROC-AUC is a more important metric to represent the performance of a prediction/classification model. The ROC-AUC clearly demonstrates the ability of different ML algorithms to accurately detect various malicious attacks in network traffic. The ROC-AUC score is a measure of the diagnostic ability of the classifier model. ROC is a probability curve, while AUC represents the measure of separability accuracy rate under various threshold tunings and rank features. The higher the percentage of accuracy (i.e., more AUC), the better the model prediction and/or classification performance. One of the main advantages of the ROC is to compare between algorithms as Figure 12 shows. The ROC-AUC curve was generated to outline multiple classes. Therefore, it is preferable to use the ROC-AUC first to understand which algorithms are the most accurate toward determining (i.e., down selecting) the prime ML algorithms before embarking on building a detection/prediction model. Figure 11 and Figure 12, and Table 6 answer our experimental question, therefore, to achieve a high rate of accuracy. In this way, we used various threshold tunings to obtain high accuracy and performance rates. Furthermore, the challenges in cybersecurity issues are related to dealing with unbalanced categories. In particular, IoT ecosystem generates locally in an individual sensor/node or globally in a centralized location enormous data, which results in a general, imbalance dataset. An imbalanced dataset problem prevents achieving high accuracy in shallow machine learning algorithms due to the different weights in each class.

## 7. Conclusions and Future Direction

The results from our comprehensive assessment for using DL anomaly-based IDSs in the IoT environment are presented. Based on the reviewed approaches, DL-based techniques can provide a more effective method for monitoring and detecting malicious nefarious intrusions. Most of the approaches provided high true-negative rates. While some approaches in “IoT-tailored” anomaly-based detection systems that use deep learning gave better detection rates for certain attacks, others were effective at detecting other atypical disruptions. We also observed that most approaches focused on the classification or detection phase in employing DL techniques. None of the reviewed approaches used DL for feature extraction in IoT-tailored anomaly-based detection systems. However, with the variety of data produced in IoT environments, applying hand-crafted manual feature selection is time consuming. Instead, we claim that learned features extracted with some DL techniques would improve the performance of anomaly-based IDS approaches.

There are feature-extraction DL architectures adopted in many domains, such as image recognition, image processing, image retrieval, etc. [88]. Therefore, it is necessary to investigate the adoption of an end-to-end DL model for anomaly-based IDSs in the IoT environment. For instance, the researchers in [31] used CNN-based features for an anomaly-detection model. In addition, well-known DL architectures, such as CNN, RNN, or LSTM, have rarely been utilized (i.e., investigated) for anomaly-based IoT detection systems. Moreover, to the best of our knowledge, there is no single study that refutes the employment of such DL approaches. Hence, in the future, investigations that involve the use of such popular DL approaches to detect anomalies in the IoT environment are a promising avenue to study and understand the potential impact they may bring to this increasingly enormous IoT assurance problem.

In this paper, therefore, we compared the DL anomaly-based IDSs approaches that have been used from published research accounted for in the matrix shown in Table 7. Table 7 depicts detection accuracy, resource consumption, false-positive rate, real-time capability, scalability, flexibility, and robustness of each approach. Table 7 shows the criteria used in each of the previous research studies that are available in the literature, where the check (✓) indicates that the criterion is discussed in the research, and “☓” indicates otherwise. We clearly observed that most previous works do not include both prediction and classification together. We also observed that the classification accuracy criteria are receiving more attention from researchers, contrary to flexibility and robustness criteria, which do not get much attention. We wanted to define other meanings and not just (☓, ✓), for example, good/bad performance/criteria; however, since it depends on personal opinions, authors preferred to use (☓, ✓) to give better proportionality between the criteria. In addition, the extracting features approach did not pay attention to the more profound studying. Most of these approaches have been evaluated against the accuracy of detecting anomalies (e.g., intrusions) in IoT. In [105], the approach was reported to have achieved an accuracy of 70% and 60% FPR. The approach in [80] showed a significant variation in accuracy between the attack scenarios, with a margin between 40–50%. The authors in [110] evaluated their work against simulated IoT networks, demonstrating 99% accuracy, while in [111], the approach was able to recover categorical features with an accuracy of 99%. With the other two remaining approaches, in [83], an overall accuracy of detection increased from around 96% to over 99% with the increase in layers (i.e., nodes), and the approach [113] showed a 99.5% accuracy for simulated networks and 98.47% accuracy when implemented on an experimental IoT testbed. These results show that there are differences, as might be expected, that result in a range of accuracies reported.

There are two issues that can be highlighted based on this review. First, the resource-constrained nature of IoT devices is one of the main limitations that make the adoption of IDSs in IoT environments challenging. Conventional IDSs cannot be implemented in the IoT environment because of their computational cost. Such approaches require a heavy number of resources (e.g., memory/storage for data-classification purposes) not typically available in such environments. To that end, lightweight IDSs that need fewer resources are required. The second issue concerns centralized data analysis employed by IDSs, which is not fundamental in IoT. In this sense, anomaly-based IDSs are more suitable to the distributed nature of IoT because they actually can minimize communication overhead, reduce network traffic, and are a less invasive cure to detecting malicious intrusions.

Implementing distributed anomaly-based IDSs can be investigated as a solution [12,14,15,24,60,69,71,72,74,75,76,77,81,82,99] to providing a less invasive IDS. Regarding detection techniques, such as the anomaly-based IDSs, the preparation and testing time needed to accomplish the normal behavior of networks is high. A huge dataset is needed for training such IDSs to accomplish the normal behavior of the IoT network. Designing an IDS that can handle these problems is challenging. DL techniques using anomaly-based IDSs are a promising avenue for future research. Some other characteristics that future IoT-IDSs may have included a real-time approach with safe routing processes. We conclude that more attention must be paid to building a strong IDS as an intelligent system based on strong DL algorithms. This would help to prevent the onslaught of attacks to the victim (IoT device) and to improve the accuracy of detection.

In summary, this manuscript documents a review of anomaly-based IDS systems used in IoT, deep learning techniques, intrusion detection systems, the IoT security, and various IoT attacks, including the various common architectures found in the IoT ecosystem. Furthermore, this study creates a novel mapping between various architectures to match them in term of structure, functionality, and security. The deep learning approaches for IDSs included supervised, unsupervised, and hybrid models. Specifically, this paper analyzed several deep learning approaches to anomaly-based IDSs in the literature. The deep learning approaches used in these anomaly-based IDS systems involved a proposed detection model or a comparative analysis of the various (published) approaches. Different approaches used different performance metrics without accounting for the type of network protocols and inherent weakness of the various IoT devices. Likewise, common datasets utilized in the IDS systems used in IoT environments were highlighted. The reviewed anomaly-based IDS systems studies either used their own data or the NLS-KDD dataset. Deep learning-based anomaly detection used in IoT continues to be an active area of research, and our future studies plan to extend and update this review as more sophisticated techniques are developed.

## Figures and Tables

**Table 1:** Comparison of Intrusion Detection Systems (IDSs) properties.

Survey Area	Survey Content	Design	Focused Domain	Attacks	ML/DL Methods	Type of Experiment	Reference
IoT Vision:	IoT application	six-layer IoT architecture	IoT architecture	√	-	-	[4] 2014
IDS in IoT	IDS in IoT	-	IDS	√	ML	Statistical analysis	[7] 2018
		Five research questions	IDS	√	-	-	[8] 2019
	IDS	-	IDS	√	-	-	[9] 2018
	IoT architecture IoT security	IoT-IDS architecture	IDS	√	-	-	[6] 2018
IoT Security-Based Data Analysis	IoT security	Five-layer IoT architecture	IoT Security	√	ML	-	[11] 2020
IoT Architectures and Applications	IoT architectures	IoT taxonomy	Five-layer architecture	-	-	-	[3] 2017
IoT-Based Info of Things		-	IoT architecture	-	-	-	[2] 2013
IoT Architecture		IoT survey taxonomy	IoT architecture, Protocols and security Privacy	-	-	-	[13] 2020
Attacks	Attacks	-	RPL and 6LoWPAN in IoT	√	-	-	[5] 2015
NIDS for IoT	NIDS	IoT threats classification	Three-layer architecture, IoT threats NIDS	√	ML	Statistical analysis	[10] 2019
ML/DL Methods for IoT Security	ML/DL in IoT IoT threats	IoTsys and threats ML/DL taxonomy	IoT security Six-layer IoT architecture	√	ML/DL	-	[12] 2020

**Table 2:** IoT architecture, attacks, and security requirements.

Layers	Attacks	Security Requirements
Application	Data accessibility and authentication, Data privacy and identity, Dealing with availability	Privacy protection, Authentication, Information security management,
Network	Man-in-the-middle, Denial of service, Eavesdropping/Sniffing, Routing attack.	Authentication, Communication security, Key management, Routing security, Intrusion detection,
Perception	Node capture, Denial of service, Denial of sleep, Distributed denial of service, Fake node/Sybil, Replay, Side channel, Mass node authentication,	Data confidentiality, Lightweight encryption, Key management, Authentication.

**Table 3:** Advantage and disadvantage of Anomaly-based IDS detection techniques.

Techniques	Advantages	Disadvantages
Machine Learning	High detection accuracy	Requires training data
	Suitable for massive data volumes	Long training time
Data Mining	Models are created automatically	Misuse of information
		Security issues
	Suitable for online datasets	Depends on complex algorithms
	Applicable in various environments	Privacy issues
Payload Model	High detection accuracy for known attacks	Privacy issues
		Long processing time
Statistical Model	Suitable for online datasets	Based on historical behavior
	System simplicity	Detection accuracy depends on statistical and arithmetic ops
		Limitation with large dataset

**Table 4:** Comparison of deep learning techniques that are used for anomaly-based IDSs in IoT.

Ref.	DL Approach	Strategy	Dataset	Advantage	Disadvantage
[83]	DNN	Classification	NSL-KDD	Real-time, high accuracy, utilizing a fog computing	Needs more time and resources
[101]	Vector-CNN	Feature extraction using VCN and Classification using FCN	Bot-IoT	Scalable detection, less time	Works better with selected features
[102]	LSTM	Classification	Own real data and ADFA-LD	Real-time, high accuracy	Needs more time
[103]	LSTM	Classification	Own real data	Real-time, high accuracy	Needs more time, more resources
[105]	Auto-Encoder	Classification	Own real data	Self-adoptive, low false neg	Does not match large scale data
[106]	Hybrid -DNN	Classification, prediction	KDD99 and NSL-KDD datasets	Suitable for heterogeneous environments, comparison with ML techniques	Average accuracy, fewer training samples
[107]	LSTM	Prediction, regression	Sensory data	Prediction accuracy, real-world data, comparison with other results	Does not handle inner-relations of data
[108]	LSTM	Classification	CIDDS	Comparison with ML techniques	Not suitable for Zero-day attacks, not suitable for heterogeneous env.
[109]	Simple neural network	Classification	NSL-KDD	Low processing time, high detection accuracy, scalability	Not real-time capable, non-suitable for zero-day attack
[110]	Simple Neural network	Classification	Own real data	Real-time usage, high detection accuracy,	Needs more time for good efficiency
[111]	ID-CVAE	Classification	NSL-KDD	Less complex, low latency	High false positive, needs more resource for training
[112]	GA and DBN	Classification	NSL-KDD	High accuracy, self-adaption	Lack experiments on the test-set, does not match large scale data
[113]	DNN	Classification	Own real data	Real time detection	needs more time
[114]	BiRNN, LSTM	Malware analysis, Feature extraction	IoT application, own real data	Comparative accuracy with various ML techniques, self-training, high accuracy, multiple models	Not suitable for complex patterns, computational-complexity-learning, random initialization
[115]	GRU, Random forest	Feature extraction, Classification	KDD-99	Multi-layered IDS, Comparisons with existing IDSs	Outdated dataset, stationary model
[116]	Vector-CNN	Feature extraction	NSL-KDD dataset	Needs minimum data	Need more resources as data increase

**Table 5:** Attack types selected from the BoT-IoT dataset.

Main Category Attack	Subcategory Attack	Training and Testing
DDoS/DoS	UDP	1,981,230
	TCP	1,593,180
	HTTP	2474
Total: DDoS/DoS		3,576,884
Reconnaissance	Service Scanning	73,168
	OS_ Fingerprinting	17,914
Normal	Normal	477
Theft	Keylogging	73
Total: theft	Data Exfiltration	6
		79
Total		3,668,522

**Table 6:** Performance of various ML algorithms relative to the various attack type and normal.

+	LR	SVM	DT	ANN
DoS-S. F	100%	100%	100%	100%
M.ARP.S	93%	92%	99%	98%
M.A. F	87%	87%	81%	90%
M.HTTP.F	87%	87%	81%	90%
M. Host. B. force	87%	87%	100%	98%
M.UDP.F	95%	95%	93%	96%
Normal	97%	97%	100%	100%
S.H. P	86%	88%	96%	95%
S.P. OS	90%	91%	98%	97%

**Table 7:** A comparison of deep anomaly-based IDS approaches in IoT.

Scheme	Classification Accuracy	Prediction Accuracy	Resource Consumption	False-Positive Rate	Real-Time Capability	Scalability	Flexibility	Robustness
[83]	✓	☓	☓	✓	☓	✓	☓	☓
[101]	✓	✓	☓	✓	✓	✓	☓	☓
[102]	✓	☓	☓	✓	☓	☓	☓	✓
[103]	✓	✓	☓	✓	☓	☓	✓	☓
[105]	☓	☓	✓	✓	☓	☓	☓	☓
[106]	✓	✓	✓	✓	✓	☓	☓	✓
[107]	☓	✓	☓	☓	✓	✓	☓	✓
[108]	✓	☓	☓	✓	☓	✓	✓	☓
[109]	✓	☓	✓	✓	☓	✓	☓	☓
[110]	✓	☓	☓	✓	☓	✓	☓	☓
[111]	✓	☓	☓	☓	✓	☓	✓	☓
[112]	✓	☓	✓	✓	☓	☓	✓	☓
[113]	☓	☓	✓	✓	✓	✓	☓	☓
[114]	✓	☓	☓	☓	✓	✓	☓	✓
[115]	✓	☓	✓	✓	☓	✓	✓	☓
[116]	✓	✓	☓	✓	✓	☓	☓	✓

## Data Availability

Not applicable.

## References

[1] Holst A. (2018). Number of Connected Devices Worldwide 2030.

[2] Said O., Masud M. (2013). Towards Internet of Things: Survey and Future Vision. Int. J. Comput. Netw..

[3] Sethi P., Sarangi S.R. (2017). Internet of Things: Architectures, Protocols, and Applications. J. Electr. Comput. Eng..

[4] Borgia E. (2014). The Internet of Things vision: Key features, applications and open issues. Comput. Commun..

[5] Pongle P., Chavan G. (2015). A survey: Attacks on RPL and 6LoWPAN in IoT. Proceedings of the 2015 International Conference on Pervasive Computing (ICPC).

[6] Benkhelifa E., Welsh T., Hamouda W. (2018). A Critical Review of Practices and Challenges in Intrusion Detection Systems for IoT: Toward Universal and Resilient Systems. IEEE Commun. Surv. Tutor..

[7] Elrawy M.F., Awad A.I., Hamed H.F.A. (2018). Intrusion detection systems for IoT-based smart environments: A survey. J. Cloud Comput..

[8] Hajiheidari S., Wakil K., Badri M., Navimipour N.J. (2019). Intrusion detection systems in the Internet of things: A comprehensive investigation. Comput. Netw..

[9] Santos L., Rabadao C., Goncalves R. (2018). Intrusion detection systems in Internet of Things: A literature review. Proceedings of the 2018 13th Iberian Conference on Information Systems and Technologies (CISTI).

[10] Chaabouni N., Mosbah M., Zemmari A., Sauvignac C., Faruki P. (2019). Network Intrusion Detection for IoT Security Based on Learning Techniques. IEEE Commun. Surv. Tutor..

[11] Mrabet H., Belguith S., Alhomoud A., Jemai A. (2020). A Survey of IoT Security Based on a Layered Architecture of Sensing and Data Analysis. Sensors.

[12] Al-Garadi M.A., Mohamed A., Al-Ali A.K., Du X., Ali I., Guizani M. (2020). A Survey of Machine and Deep Learning Methods for Internet of Things (IoT) Security. IEEE Commun. Surv. Tutor..

[13] Sobin C.C. (2020). A Survey on Architecture, Protocols and Challenges in IoT. Wirel. Pers. Commun..

[14] Hassija V., Chamola V., Saxena V., Jain D., Goyal P., Sikdar B. (2019). A Survey on IoT Security: Application Areas, Security Threats, and Solution Architectures. IEEE Access.

[15] Hindy H., Brosset D., Bayne E., Seeam A.K., Tachtatzis C., Atkinson R., Bellekens X. (2020). A Taxonomy of Network Threats and the Effect of Current Datasets on Intrusion Detection Systems. IEEE Access.

[16] Koroniotis N., Moustafa N., Sitnikova E., Turnbull B. (2019). Towards the development of realistic botnet dataset in the Internet of Things for network forensic analytics: Bot-IoT dataset. Future Gener. Comput. Syst..

[17] Ullah I., Mahmoud Q.H., Goutte C., Zhu X. (2020). A Scheme for Generating a Dataset for Anomalous Activity Detection in IoT Networks. Advances in Artificial Intelligence.

[18] Ferrag M.A., Maglaras L., Moschoyiannis S., Janicke H. (2019). Deep learning for cyber security intrusion detection: Approaches, datasets, and comparative study. J. Inf. Secur. Appl..

[19] Aldweesh A., Derhab A., Emam A. (2019). Deep learning approaches for anomaly-based intrusion detection systems: A survey, taxonomy, and open issues. Knowl. Based Syst..

[20] Liu H., Lang B. (2019). Machine Learning and Deep Learning Methods for Intrusion Detection Systems: A Survey. Appl. Sci..

[21] Ghosh A.K., Wanken J., Charron F. (1998). Detecting anomalous and unknown intrusions against programs. Proceedings of the 14th Annual Computer Security Applications Conference.

[22] García-Teodoro P., Díaz-Verdejo J., Maciá-Fernández G., Vázquez E. (2009). Anomaly-based network intrusion detection: Techniques, systems and challenges. Comput. Secur..

[23] Abduvaliyev A., Pathan A.-S.K., Zhou J., Roman R., Wong L. (2013). On the Vital Areas of Intrusion Detection Systems in Wireless Sensor Networks. IEEE Commun. Surv. Tutor..

[24] Le A., Loo J., Chai K.K., Aiash M. (2016). A Specification-Based IDS for Detecting Attacks on RPL-Based Network Topology. Information.

[25] Bostani H., Sheikhan M. (2017). Hybrid of anomaly-based and specification-based IDS for Internet of Things using unsupervised OPF based on MapReduce approach. Comput. Commun..

[26] Li W., Tug S., Meng W., Wang Y. (2019). Designing collaborative blockchained signature-based intrusion detection in IoT environments. Future Gener. Comput. Syst..

[27] Roesch M. Snort–Lightweight Intrusion Detection for Networks. Proceedings of the LISA ’99: 13th Systems Administration Conference.

[28] Snort—Network Intrusion Detection & Prevention System. https://www.snort.org/.

[29] Shah S.A.R., Issac B. (2018). Performance comparison of intrusion detection systems and application of machine learning to Snort system. Future Gener. Comput. Syst..

[30] Gómez J.A., Arévalo J., Paredes R., Nin J. (2018). End-to-end neural network architecture for fraud scoring in card payments. Pattern Recognit. Lett..

[31] Napoletano P., Piccoli F., Schettini R. (2018). Anomaly Detection in Nanofibrous Materials by CNN-Based Self-Similarity. Sensors.

[32] Hinton G.E. (2009). Deep Belief Networks. Scholarpedia.

[33] Giles C.L., Kuhn G.M., Williams R.J. (1994). Dynamic recurrent neural networks: Theory and applications. IEEE Trans. Neural Netw..

[34] Schuster M., Paliwal K.K. (1997). Bidirectional recurrent neural networks. IEEE Trans. Signal Process..

[35] Hochreiter S., Schmidhuber J. (1997). Long Short-Term Memory. Neural Comput..

[36] Cho K., van Merrienboer B., Bahdanau D., Bengio Y. (2014). On the Properties of Neural Machine Translation: Encoder-Decoder Approaches. arXiv.

[37] Salakhutdinov R., Hinton G. Deep Boltzmann Machines. Proceedings of the Machine Learning Research: Artificial Intelligence and Statistics.

[38] Hinton G.E., Salakhutdinov R.R. (2006). Reducing the Dimensionality of Data with Neural Networks. Science.

[39] Das S., Venugopal D., Shiva S., Sheldon F.T. Empirical evaluation of the ensemble framework for feature selection in ddos attack. Proceedings of the 2020 7th IEEE International Conference on Cyber Security and Cloud Computing (CSCloud)/2020 6th IEEE International Conference on Edge Computing and Scalable Cloud (EdgeCom).

[40] Li D., Deng L., Lee M., Wang H. (2019). IoT data feature extraction and intrusion detection system for smart cities based on deep migration learning. Int. J. Inf. Manag..

[41] Trappe W., Howard R., Moore R.S. (2015). Low-Energy Security: Limits and Opportunities in the Internet of Things. IEEE Secur. Priv. Mag..

[42] Hernandez G., Arias O., Buentello D., Jin Y. (2015). Smart Nest Thermostat: A Smart Spy in Your Home.

[43] Mouaatamid O.E., Lahmer M., Belkasmi M. (2016). Internet of Things Security: Layered classification of attacks and possible Countermeasures. Electron. J. Inf. Technol..

[44] Smadi A., Ajao B., Johnson B., Lei H., Chakhchoukh Y., Abu Al-Haija Q. (2021). A Comprehensive Survey on Cyber-Physical Smart Grid Testbed Architectures: Requirements and Challenges. Electronics.

[45] El-Hajj M., Fadlallah A., Chamoun M., Serhrouchni A. (2019). A Survey of Internet of Things (IoT) Authentication Schemes. Sensors.

[46] Wazid M., Das A.K., Hussain R., Succi G., Rodrigues J.J. (2019). Authentication in cloud-driven IoT-based big data environment: Survey and outlook. J. Syst. Archit..

[47] Azure IoT Reference Architecture Update. https://azure.microsoft.com/en-us/blog/azure-iot-reference-architecture-update/.

[48] Sap-IoT-Reference-Architecture. https://www.intel.com/content/dam/www/public/us/en/documents/reference-architectures/sap-iot-reference-architecture.pdf.

[49] IoT-Platform-Solution-Brief. https://www.intel.com/content/dam/www/public/us/en/documents/solution-briefs/iot-platform-solution-brief.pdf.

[50] Miloslavskaya N., Nikiforov A., Plaksiy K., Tolstoy A. (2019). Standardization Issues for the Internet of Things.

[51] Lin J., Yu W., Zhang N., Yang X., Zhang H., Zhao W. (2017). A Survey on Internet of Things: Architecture, Enabling Technologies, Security and Privacy, and Applications. IEEE Internet Things J..

[52] Vishwakarma R., Jain A.K. (2019). A survey of DDoS attacking techniques and defence mechanisms in the IoT network. Telecommun. Syst..

[53] Rehman S.U., Singh P., Manickam S., Praptodiyono S. (2020). Towards Sustainable IoT Ecosystem. Proceedings of the 2020 2nd International Conference on Industrial Electrical and Electronics (ICIEE).

[54] Wu M., Lu T.J., Ling F.Y., Sun J., Du H.Y. Research on the architecture of Internet of Things. Proceedings of the 2010 3rd International Conference on Advanced Computer Theory and Engineering(ICACTE).

[55] Fang S., Da Xu L., Zhu Y., Ahati J., Pei H., Yan J., Liu Z. (2014). An Integrated System for Regional Environmental Monitoring and Management Based on Internet of Things. IEEE Trans. Ind. Inform..

[56] IoT-Overview. ITU-T Recommendations, ITU-T Y.4000/Y.2060 (06/2012), Overview of the Internet of Things. http://handle.itu.int/11.1002/1000/11559.

[57] Intel^®^ Network Builders—Network Transformation Technologies, NFV/SDN. Intel^®^ Network Builders. https://networkbuilders.intel.com/.

[58] Das S., Ashrafuzzaman M., Sheldon F.T., Shiva S. (2020). Network Intrusion Detection using Natural Language Processing and Ensemble Machine Learning. Proceedings of the 2020 IEEE Symposium Series on Computational Intelligence (SSCI).

[59] Camacho J., Theron R., Garcia-Gimenez J.M., Macia-Fernandez G., Garcia-Teodoro P. (2019). Group-Wise Principal Component Analysis for Exploratory Intrusion Detection. IEEE Access.

[60] Elrawy M.F., Awad A.I., Hamed H.F.A. (2016). Flow-based features for a robust intrusion detection system targeting mobile traffic. Proceedings of the 2016 23rd International Conference on Telecommunications (ICT).

[61] Summerville D.H., Zach K.M., Chen Y. (2015). Ultra-lightweight deep packet anomaly detection for Internet of Things devices. Proceedings of the 2015 IEEE 34th International Performance Computing and Communications Conference (IPCCC).

[62] Abhishek N.V., Lim T.J., Sikdar B., Tandon A. An Intrusion Detection System for Detecting Compromised Gateways in Clustered IoT Networks. Proceedings of the 2018 IEEE International Workshop Technical Committee on Communications Quality and Reliability (CQR).

[63] Arrington B., Barnett L., Rufus R., Esterline A. (2016). Behavioral Modeling Intrusion Detection System (BMIDS) Using Internet of Things (IoT) Behavior-Based Anomaly Detection via Immunity-Inspired Algorithms. Proceedings of the 2016 25th International Conference on Computer Communication and Networks (ICCCN).

[64] Deng L., Li D., Yao X., Cox D., Wang H. (2019). Mobile network intrusion detection for IoT system based on transfer learning algorithm. Clust. Comput..

[65] Makani R., Reddy B. (2018). Taxonomy of Machine Leaning Based Anomaly Detection and its suitability. Procedia Comput. Sci..

[66] Tsai C.-F., Hsu Y.-F., Lin C.-Y., Lin W.-Y. (2009). Intrusion detection by machine learning: A review. Expert Syst. Appl..

[67] Feng W., Zhang Q., Hu G., Huang J.X. (2014). Mining network data for intrusion detection through combining SVMs with ant colony networks. Future Gener. Comput. Syst..

[68] Alseiari F.A.A., Aung Z. (2015). Real-time anomaly-based distributed intrusion detection systems for advanced Metering Infrastructure utilizing stream data mining. Proceedings of the 2015 International Conference on Smart Grid and Clean Energy Technologies (ICSGCE).

[69] Xu C., Chen S., Su J., Yiu S.M., Hui L.C.K. (2016). A Survey on Regular Expression Matching for Deep Packet Inspection: Applications, Algorithms, and Hardware Platforms. IEEE Commun. Surv. Tutor..

[70] Weller-Fahy D.J., Borghetti B.J., Sodemann A.A. (2015). A Survey of Distance and Similarity Measures Used Within Network Intrusion Anomaly Detection. IEEE Commun. Surv. Tutor..

[71] Raza S., Wallgren L., Voigt T. (2013). SVELTE: Real-time intrusion detection in the Internet of Things. Ad Hoc Netw..

[72] Fu R., Zheng K., Zhang D., Yang Y. (2011). An Intrusion Detection Scheme Based on Anomaly Mining in Internet of Things.

[73] Ding Y., Zhou X.-W., Cheng Z.-M., Lin F.-H. (2013). A Security Differential Game Model for Sensor Networks in Context of the Internet of Things. Wirel. Pers. Commun..

[74] Chen P.-Y., Cheng S.-M., Chen K.-C. (2014). Information Fusion to Defend Intentional Attack in Internet of Things. IEEE Internet Things J..

[75] Rajasegarar S., Gluhak A., Imran M.A., Nati M., Moshtaghi M., Leckie C., Palaniswami M. (2014). Ellipsoidal neighbourhood outlier factor for distributed anomaly detection in resource constrained networks. Pattern Recognit..

[76] Ham H.-S., Kim H.-H., Kim M.-S., Choi M.-J. (2014). Linear SVM-Based Android Malware Detection for Reliable IoT Services. J. Appl. Math..

[77] Wang J., Kuang Q., Duan S. (2015). A new online anomaly learning and detection for large-scale service of Internet of Thing. Pers. Ubiquitous Comput..

[78] Pongle P., Chavan G. (2015). Real Time Intrusion and Wormhole Attack Detection in Internet of Things. Int. J. Comput. Appl..

[79] Zhang C., Green R.C. (2015). Communication security in internet of thing: Preventive measure and avoid DDoS attack over IoT network. Simul. Ser..

[80] Cervantes C., Poplade D., Nogueira M., Santos A. (2015). Detection of sinkhole attacks for supporting secure routing on 6LoWPAN for Internet of Things. Proceedings of the 2015 IFIP/IEEE International Symposium on Integrated Network Management (IM).

[81] Rahman S., Al Mamun S., Ahmed M.U., Kaiser M.S. (2016). PHY/MAC layer attack detection system using neuro-fuzzy algorithm for IoT network. Proceedings of the 2016 International Conference on Electrical, Electronics, and Optimization Techniques (ICEEOT).

[82] Albulayhi K., Sheldon F.T. (2021). An Adaptive Deep-Ensemble Anomaly-Based Intrusion Detection System for the Internet of Things. Proceedings of the 2021 IEEE World AI IoT Congress (AIIoT).

[83] Diro A.A., Chilamkurti N. (2018). Distributed attack detection scheme using deep learning approach for Internet of Things. Future Gener. Comput. Syst..

[84] Javaid A., Niyaz Q., Sun W., Alam M. (2016). A Deep Learning Approach for Network Intrusion Detection System. Proceedings of the 9th EAI International Conference on Bio-Inspired Information and Communications Technologies (Formerly BIONETICS).

[85] Tang T.A., Mhamdi L., McLernon D., Zaidi S.A.R., Ghogho M. Deep learning approach for Network Intrusion Detection in Software Defined Networking. Proceedings of the 2016 International Conference on Wireless Networks and Mobile Communications (WINCOM).

[86] Kang M.J., Kang J.W. (2016). Intrusion Detection System Using Deep Neural Network for In-Vehicle Network Security. PLoS ONE.

[87] Chalapathy R., Chawla S. (2019). Deep Learning for Anomaly Detection: A Survey. arXiv.

[88] Schmidhuber J. (2015). Deep learning in neural networks: An overview. Neural Netw..

[89] Abbass W., Bakraouy Z., Baïna A., Bellafkih M. (2018). Classifying IoT security risks using Deep Learning algorithms. Proceedings of the 2018 6th International Conference on Wireless Networks and Mobile Communications (WINCOM).

[90] Bengio Y. (2009). Learning Deep Architectures for AI.

[91] Zaza A.M., Kharroub S.K., Abualsaud K. (2020). Lightweight IoT Malware Detection Solution Using CNN Classification. Proceedings of the 2020 IEEE 3rd 5G World Forum (5GWF).

[92] More S., Singla J., Verma S., Ghosh U., Rodrigues J.J., Hosen A.S., Ra I.-H. (2020). Security Assured CNN-Based Model for Reconstruction of Medical Images on the Internet of Healthcare Things. IEEE Access.

[93] Liao C.-H., Shuai H.-H., Wang L.-C. (2019). RNN-Assisted Network Coding for Secure Heterogeneous Internet of Things With Unreliable Storage. IEEE Internet Things J..

[94] Alom M.Z., Taha T.M. (2017). Network intrusion detection for cyber security using unsupervised deep learning approaches. Proceedings of the 2017 IEEE National Aerospace and Electronics Conference (NAECON).

[95] Ackley D.H., Hinton G.E., Sejnowski T.J. (1985). A learning algorithm for boltzmann machines. Cogn. Sci..

[96] Fissore G., Decelle A., Furtlehner C., Han Y. (2019). Robust Multi-Output Learning with Highly Incomplete Data via Restricted Boltzmann Machines. arXiv.

[97] Elsaeidy A., Munasinghe K.S., Sharma D., Jamalipour A. (2019). Intrusion detection in smart cities using Restricted Boltzmann Machines. J. Netw. Comput. Appl..

[98] Hiromoto R.E., Haney M., Vakanski A. A secure architecture for IoT with supply chain risk management. Proceedings of the 2017 9th IEEE International Conference on Intelligent Data Acquisition and Advanced Computing Systems: Technology and Applications (IDAACS).

[99] Chen Z., Fu A., Zhang Y., Liu Z., Zeng F., Deng R.H. (2020). Secure Collaborative Deep Learning against GAN Attacks in the Internet of Things. IEEE Internet Things J..

[100] Marín G., Casas P., Capdehourat G. (2018). RawPower: Deep Learning Based Anomaly Detection from Raw Network Traffic Measurements.

[101] Ng B.A., Selvakumar S. (2020). Anomaly detection framework for Internet of things traffic using vector convolutional deep learning approach in fog environment. Future Gener. Comput. Syst..

[102] Wang X., Lu X. (2020). A Host-Based Anomaly Detection Framework Using XGBoost and LSTM for IoT Devices. Wirel. Commun. Mob. Comput..

[103] Wu D., Jiang Z., Xie X., Wei X., Yu W., Li R. (2020). LSTM Learning With Bayesian and Gaussian Processing for Anomaly Detection in Industrial IoT. IEEE Trans. Ind. Inform..

[104] O’Shea T.J., Clancy T.C., McGwier R.W. (2016). Recurrent Neural Radio Anomaly Detection. arXiv.

[105] Luo T., Nagarajan S.G. Distributed Anomaly Detection Using Autoencoder Neural Networks in WSN for IoT. Proceedings of the 2018 IEEE International Conference on Communications (ICC).

[106] Ma T., Wang F., Cheng J., Yu Y., Chen X. (2016). A Hybrid Spectral Clustering and Deep Neural Network Ensemble Algorithm for Intrusion Detection in Sensor Networks. Sensors.

[107] Zhang W., Guo W., Liu X., Liu Y., Zhou J., Li B., Lu Q., Yang S. (2018). LSTM-Based Analysis of Industrial IoT Equipment. IEEE Access.

[108] Althubiti S.A., Jones E.M., Roy K. (2018). LSTM for Anomaly-Based Network Intrusion Detection. Proceedings of the 2018 28th International Telecommunication Networks and Applications Conference (ITNAC).

[109] Pamukov M.E., Poulkov V.K., Shterev V.A. (2018). Negative Selection and Neural Network Based Algorithm for Intrusion Detection in IoT. Proceedings of the 2018 41st International Conference on Telecommunications and Signal Processing (TSP).

[110] Hodo E., Bellekens X., Hamilton A., Dubouilh P.-L., Iorkyase E., Tachtatzis C., Atkinson R. Threat analysis of IoT networks using artificial neural network intrusion detection system. Proceedings of the 2016 International Symposium on Networks, Computers and Communications (ISNCC).

[111] Lopez-Martin M., Carro B., Sanchez-Esguevillas A., Lloret J. (2017). Conditional Variational Autoencoder for Prediction and Feature Recovery Applied to Intrusion Detection in IoT. Sensors.

[112] Zhang Y., Li P., Wang X. (2019). Intrusion Detection for IoT Based on Improved Genetic Algorithm and Deep Belief Network. IEEE Access.

[113] Thamilarasu G., Chawla S. (2019). Towards Deep-Learning-Driven Intrusion Detection for the Internet of Things. Sensors.

[114] HaddadPajouh H., Dehghantanha A., Khayami R., Choo K.-K.R. (2018). A deep Recurrent Neural Network based approach for Internet of Things malware threat hunting. Future Gener. Comput. Syst..

[115] Putchala M.K. (2017). Deep Learning Approach for Intrusion Detection System (IDS) in the Internet of Things (IoT) Network Using Gated Recurrent Neural Networks (GRU). Master’s Thesis.

[116] Jo W., Kim S., Lee C., Shon T. (2020). Packet Preprocessing in CNN-Based Network Intrusion Detection System. Electronics.

[117] NSL-KDD|Datasets|Research|Canadian Institute for Cybersecurity|UNB. https://www.unb.ca/cic/datasets/nsl.html.

[118] Beigi E.B., Jazi H.H., Stakhanova N., Ghorbani A.A. (2014). Towards effective feature selection in machine learning-based botnet detection approaches. 2014 IEEE Conference on Communications and Network Security.

[119] Shiravi A., Shiravi H., Tavallaee M., Ghorbani A.A. (2012). Toward developing a systematic approach to generate benchmark datasets for intrusion detection. Comput. Secur..

[120] Ashrafuzzaman M., Das S., Chakhchoukh Y., Shiva S., Sheldon F.T. (2020). Detecting stealthy false data injection attacks in the smart grid using ensemble-based machine learning. Comput. Secur..

